# Persistent Changes in Hormones and Growth Factors Involved in Ageing in Patients That Recovered from Severe COVID-19

**DOI:** 10.3390/diseases13070209

**Published:** 2025-07-03

**Authors:** Alice Cuchi-Cabral, André C. Palma, Guilherme A. Nogueira, Henrique Ceretta Oliveira, Suzimar F. Benato Fusco, Maria L. Moretti, Licio A. Velloso, Eliana P. Araujo

**Affiliations:** 1 School of Nursing, University of Campinas, Campinas 13083-970, SP, Brazil; alicecuchicbl@gmail.com (A.C.-C.); hceretta@unicamp.br (H.C.O.); sbenato@unicamp.br (S.F.B.F.); 2 Laboratory of Cell Signalling, Obesity and Comorbidities Research Center (OCRC), University of Campinas, Campinas 13083-970, SP, Brazil; guilherme.nogueira1989@gmail.com (G.A.N.); lavellos@unicamp.br (L.A.V.); 3 Faculty of Medical Sciences, University of Campinas, Campinas 13083-970, SP, Brazil; palma.andrecitroni@gmail.com (A.C.P.); lmoretti@unicamp.br (M.L.M.)

**Keywords:** BDNF, FGF-1, GH, IGF-1, PDGF

## Abstract

Background: The coronavirus disease-19 pandemic affected millions of people and its long-term impact on the health of survivors is under evaluation. Objectives: In this study, we hypothesized that severe coronavirus disease-19 could promote long-term changes in the blood levels of hormones and growth factors known to be involved in the regulation of ageing. Methods: We evaluated 49 patients that recovered from severe COVID-19 and compared them with matched controls that were never infected by the virus. The blood levels of growth hormone, insulin-like growth factor-1, insulin, brain-derived neurotrophic factor, nerve growth factor, oxytocin, ghrelin, platelet-derived growth factor, fibroblast growth factor-1, and transforming growth factor-beta were determined using enzyme-linked immunosorbent assays. Results: After six months of recovery, patients presented reduced blood levels of growth hormone, insulin-like growth factor-1, brain-derived neurotrophic factor, and platelet-derived growth factor. Fifteen months after, the reductions in the blood levels of all four hormones/growth factors persisted. Conclusions: Our study advances the field by identifying hormones and growth factors involved in ageing that undergo persistent changes in patients that recover from severe COVID-19. Further studies could explore the potential of the identified hormones/growth factors as therapeutic targets for the late complications and accelerated ageing that may affect patients recovering from severe COVID-19.

## 1. Introduction

Several environmental factors are known to promote accelerated ageing. Exposure to ultraviolet radiation, air pollution, smoking, and the excessive consumption of alcohol are undisputedly the factors most widely studied [1,2,3,4]. However, mounting evidence indicates that infections and inflammatory conditions may impact negatively the process of ageing [5,6].

The recent coronavirus disease-19 (COVID-19) pandemic affected more than 700 million people worldwide, resulting in the deaths of more than 7 million people. Out of the recovered patients, approximately 15% had severe disease [7], and the long-term consequences of this condition are still under intense investigation. Studies have shown that approximately 30% of patients that recovered from COVID-19 present persistent symptoms that include, but are not restricted to, fatigue, memory impairment, sleep disorder, myalgia, and dyspnea [8]. Currently, these patients are diagnosed as having post-COVID-19 syndrome or long COVID-19, a condition resulting from yet unknown mechanisms [9].

Because of the chronicity and complexity of the complications resulting from severe COVID-19, it has been proposed that it could represent a pro-ageing condition. Several factors that emerge as consequences of the disease are known to impact key functions that are fundamental for health and are impaired in ageing, such as mitochondrial function, cell growth, regeneration, and apoptosis [10].

Several factors that arise as consequences of the disease are known to impact key functions that are fundamental to health and that are impaired by ageing, such as mitochondrial function, cell growth, regeneration, and apoptosis [10]. Mongelli et al. showed a consistent increase in biological age in the post-COVID-19 population, determining an acceleration of the age delta of 10.45 ± 7.29 years (+5.25 years above the normal range) compared to the value of 3.68 ± 8.17 years for the COVID-19-free population (*p* < 0.0001) [11]. Cao et al. evaluated the DNA methylation of blood samples from 232 healthy individuals and 413 individuals with COVID-19. The epigenetic ages of each individual were determined by applying epigenetic clocks and telomere length estimators to the methylation profile of individuals [12]. The authors observed the increasing acceleration of epigenetic ageing and telomere attrition in blood samples from healthy individuals and infected individuals who developed severe COVID-19. Longitudinal analysis of DNA methylation profiles showed that epigenetic ageing accumulates in post-COVID-19 syndrome patients and can be partially reversed in late clinical phases in some individuals [13].

Moreover, inflammageing refers to chronic low-grade inflammation associated with ageing, which predisposes older adults to more severe forms of disease and persistent symptoms [12]. Chronically activated innate immune responses and impaired antiviral responses are consequences of immune system remodelling over time, described as ‘immunosenescence’ and ‘inflammageing’ [12]. These conditions are observed in COVID-19 patients who experience the worst clinical outcomes, indicating their relevance in the pathogenesis of the infection. Senescent cells acquire a pro-inflammatory phenotype known as the senescence-associated secretory phenotype (SASP), contributing to these phenomena [14]. Furthermore, telomere shortening, a marker of cellular ageing, has been associated with the severity of COVID-19. In a meta-analysis evaluating 1332 patients with severe COVID-19 and 6321 with non-severe disease, the mean difference in leukocyte telomere length was 0.39, with no clear evidence of an association between shorter telomeres and severe disease [15]. Villar-Juárez et al. also found a decreased telomere length in post-COVID-19 individuals, but no association with cognitive deficits [16]. Soares et al. found a significant reduction in sperm telomere length in COVID-19 patients, with the majority classified as asymptomatic or displaying mild disease [17].

Despite the intense efforts to advance our understanding of the long-term complications of COVID-19 and their potential impact on ageing, there are still more questions than answers. Defining circulating factors that could be associated with this process could contribute to the progress of this field.

Here, we hypothesized that the blood levels of hormones and growth factors known to impact ageing by distinct mechanisms [18] could be affected by severe COVID-19. Thus, we determined the levels of growth hormone (GH), insulin-like growth factor-1(IGF-1), insulin, brain-derived neurotrophic factor (BDNF), nerve growth factor (NGF), oxytocin, acylated ghrelin (AG), platelet-derived growth factor (PDGF), fibroblast growth factor-1 (FGF-1), and transforming growth factor-beta (TGF-β) six-months after the acute phase and fifteen months after recovery from severe COVID-19. We found that, both six and fifteen months after recovery, the levels of GH, IGF-1, BDNF, and PDGF were reduced in blood of the patients.

## 2. Materials and Methods

### 2.1. Patients and Volunteers

This is a descriptive and comparative study, carried out with secondary data and via the analysis of serum collected and stored in a biological repository. Patients were selected based on confirmed diagnosis of SARS-CoV-2 by PCR, admitted to the intensive care unit of the University Hospital of Campinas-Brazil. Patients were 18 years of age or older; O_2_ saturation in room air was 94% or less; pneumonia was diagnosed by computed tomography; consent was provided to participate in the study (by the participant or family member). We excluded pregnant or breastfeeding women, as well as patients with severe nephropathy; severe hepatopathy; HIV diagnosis; other immunodeficiencies; cancer diagnosis or hereditary angioedema; ischemic myocardial disease; and thromboembolic disease. The participants included in the study were evaluated during hospitalization for COVID-19, six months later (COVID-19/1º), and fifteen months after hospital discharge (COVID-19/2º). In each evaluation, clinical and laboratory parameters and blood samples were collected for ELISA testing. Serum samples from volunteers in the control group were acquired from the same biological repository; these were collected before the emergence of the pandemic.

The control participants were included using propensity score matching with the COVID-19 group based on age (years), body mass (kg), and BMI.

This clinical trial was registered in the Brazilian Clinical Trials Registry, Universal Trial Number (UTN) U1111-1250-1843, in June 2020.

### 2.2. Enzyme-Linked Immunosorbent Assays

Protein levels were measured using the ELISA kit (My BioSource, San Diego, CA, USA). The assay was performed following the manufacturer’s protocol. Detection was performed in a microplate reader (MicroplateReader BioTek 800 Elx, Agilent Technologies, Santa Clara, CA, USA) and measurement was immediately conducted at 450 nm. The kit specifications are as follows.
Human Insulin (INS) MBS704195Human Transforming Growth Factor Beta MBS266143Human Insulin-like Growth Factors 1 Long R3 MBS733937Human BDNF MBS2515054Human NGFMBS2509465Human Platelet-Derived Growth FactorMBS021279Human Fibroblast Growth Factor 1MBS459282Human OxytocinMBS703338Human GHMBS2513456Human Acylated ghrelinMBS166226Human SARS-Cov-2 SPIKENBP3-11407

### 2.3. Statistical Analysis

Comparisons between groups regarding quantitative characterization variables and ageing markers were performed using the unpaired Student’s *t*-test or Mann–Whitney test, according to data distribution. Data distribution was assessed using the Shapiro–Wilk test [19]. To assess associations between groups and qualitative characterization variables, Pearson’s chi-square test was applied. In cases where the assumptions of the chi-square test were not met, it was applied. We performed comparisons between the two periods, for the group of participants with COVID-19, regarding ageing markers using the paired Wilcoxon test [20]. The analyses were performed using SAS 9.4 and SPSS 23 statistical software, and a significance level of 5% was considered.

## 3. Results

### 3.1. Clinical Features of Severe COVID-19 Patients

We evaluated 49 patients that developed severe COVID-19 and 28 volunteers that had never been infected by the SARS-CoV-2. All patients in the severe COVID-19 group were treated with dexamethasone to alleviate the inflammatory process associated with the exacerbated production of cytokines, pulmonary edema, and alveolar damage. Comorbidities were treated individually according to the needs of the patients. Initially, we observed a high concentration of IL-6 and IL-1β in COVID-19 patients compared with controls, indicating disease severity in these patients (Supplementary data_Table S1). Furthermore, we performed spike measurements and found that all COVID-19 patients exhibited high serum spike concentrations at the time of admission, confirming the presence of infection. The average concentration was 238.6 ng/mL, with the lowest being 39.8 ng/mL and the highest 398.5 ng/mL, which demonstrates a correlation between the changes in the measured proteins. Additionally, at the time this data was evaluated, we did not yet have a vaccine against COVID-19; therefore, none of the patients were vaccinated. The existence of circulating spike protein may also be a consequence of vaccination [20]. There were no differences between the groups regarding age, body mass, and body mass index (BMI) (Supplementary data_Table S2).

In addition, there were no differences between the groups regarding sex, composite comorbidities, and the following isolated comorbidities: obesity, hypertension, diabetes, asthma, respiratory diseases other than asthma, and hypothyroidism. Dyslipidemia was the only comorbidity with greater prevalence in the control group (Table 1).

### 3.2. Blood Levels of Growth Hormone, Insulin-like Growth Factor-1 and Insulin in Patients Recovered from Severe COVID-19

**Figure 1:**
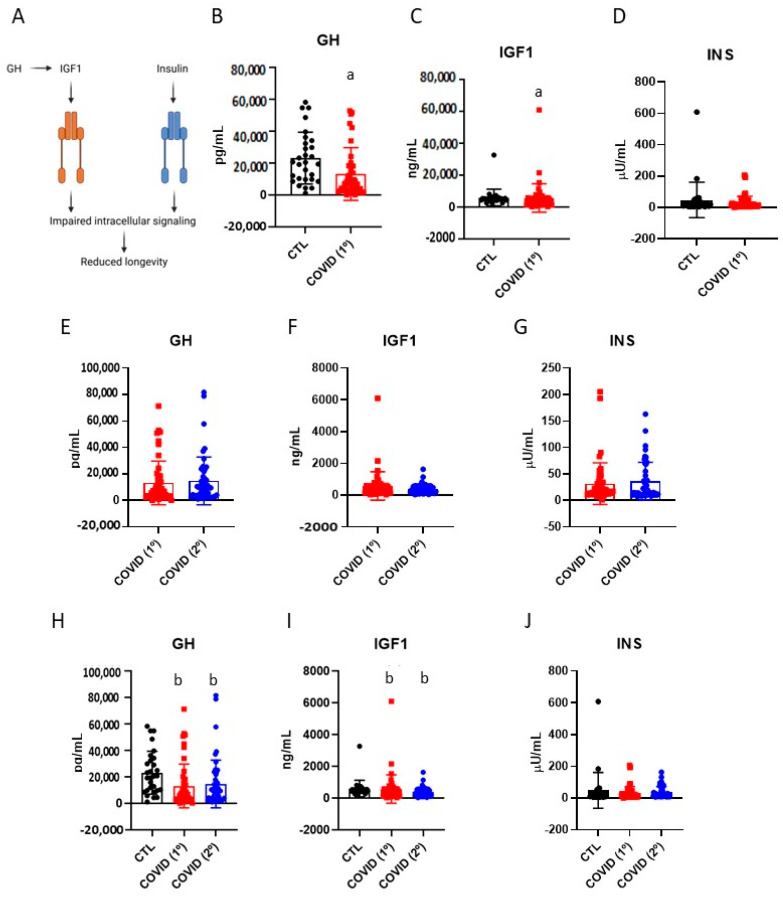
Blood levels of growth hormone, insulin-like growth factor-1, and insulin in control, six months after recovery from COVID-19 (COVID-19/1º), and fifteen months after recovery from COVID-19 (COVID-19/2º). A schematic representation of the growth hormone/insulin-like growth factor-1 and insulin system and their downstream functions (**A**). The expression of growth hormone; control vs. COVID-19/1º (**B**). The expression of insulin-like growth factor-1; control vs. COVID-19/1º (**C**). The expression of insulin; control vs. COVID-19/1º (**D**). The expression of growth hormone; COVID-19/1º vs. COVID-19/2º (**E**). The expression of insulin-like growth factor-1; COVID-19/1º vs. COVID-19/2º (**F**). The expression of insulin; COVID-19/1º vs. COVID-19/2º (**G**). The expression of growth hormone; control vs. COVID-19/1º vs. COVID-19/2º (**H**). The expression of insulin-like growth factor-1; control vs. COVID-19/1º vs. COVID-19/2º (**I**). The expression of insulin; control vs. COVID-19/1º vs. COVID-19/2º (**J**). Growth factor, GH; insulin-like growth factor-1, IGF1; insulin, INS. *n* = 28, control; *n* = 49, COVID- 19. a, *p* < 0.05 vs. control; Student’s *t* test; b, *p* < 0.05 vs. control; Wilcoxon Paired test.

The GH/IGF-1 system and insulin are well-known systemic players involved in the regulation of ageing [21] (Figure 1A). After six months of infection, patients who developed severe COVID-19 presented reduced GH (Figure 1B) and IGF-1 (Figure 1C), as compared with controls, whereas the insulin levels were not different (Figure 1D). There were no differences in the blood levels of GH, IGF-1, and insulin when comparing the samples collected after six months of disease versus those collected late after recovery (Figure 1E–G). Nevertheless, when comparing samples from controls, six months after disease, and late after recovery, the significant reductions in GH (Figure 1H) and in IGF-1 (Figure 1I) persisted, whereas insulin levels remained equal (Figure 1J).

### 3.3. Brain-Derived Neurotrophic Factor and Nerve Growth Factor in Patients Recovered from Severe COVID-19

**Figure 2:**
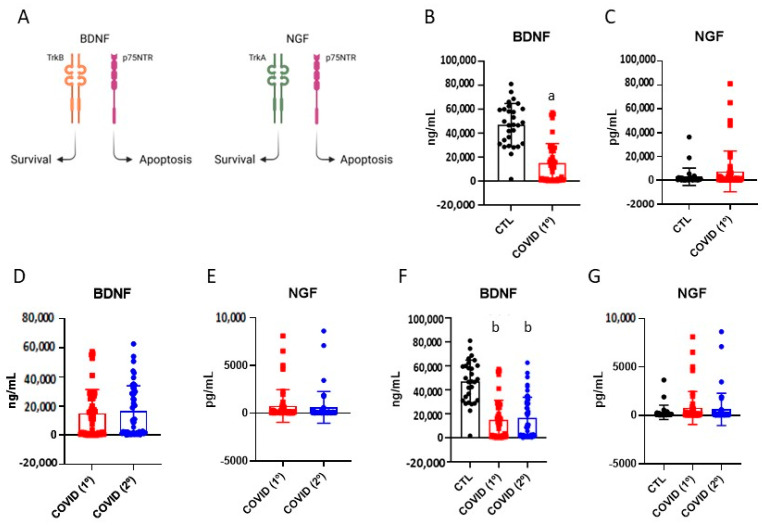
Blood levels of brain-derived neurotrophic factor and nerve growth factor in control, six months after recovery from COVID-19 (COVID-19/1º), and fifteen months after recovery from COVID-19 (COVID-19/2º). Schematic representation of brain-derived neurotrophic factor and nerve growth factor and their downstream functions (**A**). Expression of brain-derived neurotrophic factor; control vs. COVID-19/1º (**B**). Expression of nerve growth factor; control vs. COVID-19/1º (**C**). Expression of brain-derived neurotrophic factor COVID-19/1º vs. COVID-19/2º (**D**). Expression of nerve growth factor; COVID-19/1º vs. COVID-19/2º (**E**). Expression of brain-derived neurotrophic factor; control vs. COVID-19/1º vs. COVID-19/2º (**F**). Expression of nerve growth factor; control vs. COVID-19/1º vs. COVID-19/2º (**G**). BDNF; nerve growth factor, NGF; platelet-derived growth factor. *n* = 28, control; *n* = 49, COVID-19. a, *p* < 0.05 vs. control, Mann–Whitney test; b, *p* < 0.05 vs. control, Wilcoxon Paired test.

Studies showed changes in the blood levels of BDNF and NGF in humans and experimental models of ageing [22,23] (Figure 2A). After six months of COVID-19, patients presented lower blood levels of BDNF than subjects of the control group (Figure 2B). The levels of BDNF at the late stage following recovery were equal to the levels detected after six months of disease (Figure 2D), whereas the comparison of the BDNF levels in samples from subjects of the control group, after six months of disease, and late after recovery indicated that a significant reduction persisted (Figure 2F). There were no significant changes in the blood levels of NGF either after six months or during the late phase of disease (Figure 2C,E,G).

### 3.4. Oxytocin and Ghrelin Levels in Patients Recovered from Severe COVID-19

**Figure 3:**
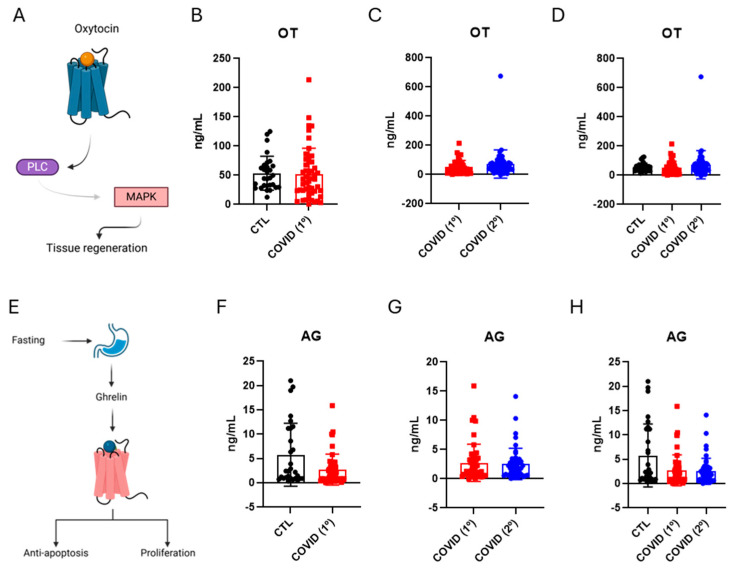
Blood levels of oxytocin and ghrelin in control six months after recovery from COVID-19 (COVID-19/1º) and fifteen months after recovery from COVID-19 (COVID-19/2º). Schematic representation of the oxytocin and ghrelin and their downstream functions (**A**). The expression of oxytocin; control vs. COVID-19/1º (**B**). Expression of oxytocin COVID-19/1º vs. COVID-19/2º (**C**). Expression of oxytocin; control vs. COVID-19/1º vs. COVID-19/2º (**D**). Schematic showing the acetylated ghrelin and their downstream functions (**E**). Expression of acetylated ghrelin; control vs. COVID-19/1º (**F**). Expression of acetylated ghrelin; COVID-19/1º vs. COVID-19/2º (**G**). Expression of ghrelin; control vs. COVID-19/1º vs. COVID-19/2º (**H**). Oxytocin, OT; ghrelin, AG. *n* = 28, control; *n* = 49, COVID-19.

Studies proposed that both oxytocin and ghrelin could be involved in the protection against ageing [24] (Figure 3A). We found no changes in the blood levels of either oxytocin (Figure 3B–D) or ghrelin (Figure 3E–G) after six months and the late phase of COVID-19.

### 3.5. Platelet-Derived Growth Factor, Fibroblast Growth Factor-1, and Transforming Growth Factor-Beta Levels in Patients Recovered from Severe COVID-19

**Figure 4:**
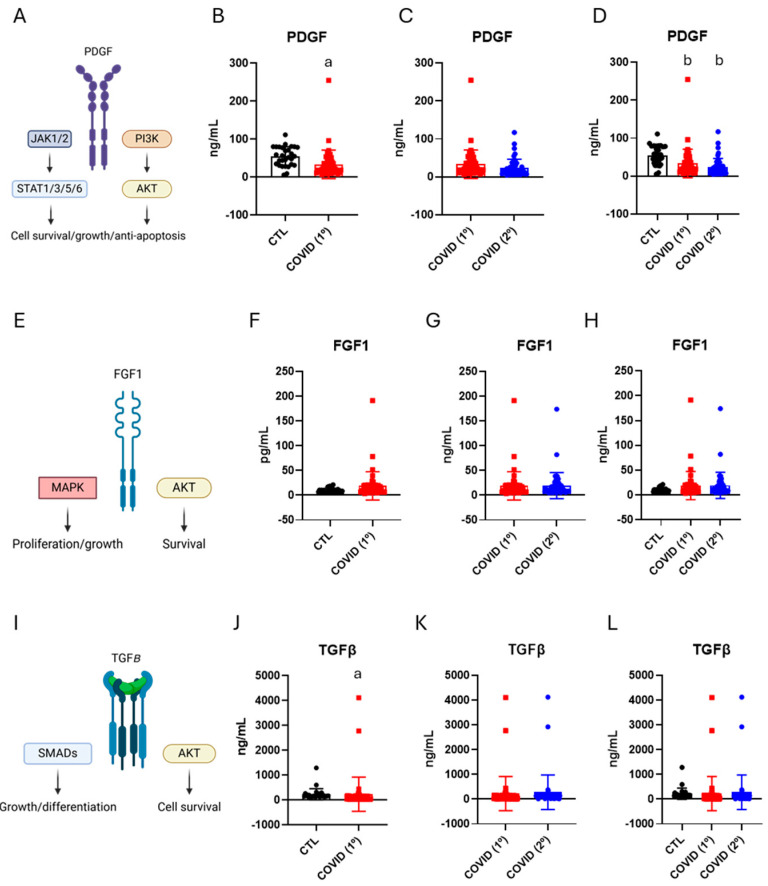
Blood levels of platelet-derived growth factor, fibroblast growth factor-1, and transforming growth factor-beta in control, six months after recovery from COVID-19 (COVID-19/1º), and fifteen months after recovery from COVID-19 (COVID-19/2º). Schematic representation of platelet-derived growth factor and their downstream functions (**A**). Expression of platelet-derived growth factor; control vs. COVID-19/1º (**B**). Expression of platelet-derived growth factor COVID-19/1º vs. COVID-19/2º (**C**). Expression of platelet-derived growth factor; control vs. COVID-19/1º vs. COVID-19/2º (**D**). Schematic showing fibroblast growth factor pathway and their downstream functions (**E**). Expression of fibroblast growth factor; control vs. COVID-19/1º (**F**). Expression of fibroblast growth factor; COVID-19/1º vs. COVID-19/2º (**G**). Expression of fibroblast growth factor pathway; control vs. COVID-19/1º vs. COVID-19/2º (**H**). Schematic showing transforming growth factor-beta pathway and their downstream functions (**I**). Expression of transforming growth factor-beta; control vs. COVID-19/1º (**J**). Expression of transforming growth factor-beta; COVID-19/1º vs. COVID-19/2º (**K**). Expression of transforming growth factor-beta; control vs. COVID-19/1º vs. COVID-19/2º (**L**). PDGF; fibroblast growth factor-1, FGF1; transforming growth factor-beta, TGF-β. *n* = 28, control; *n* = 49, COVID-19. a, *p* < 0.05 vs. control, Mann–Whitney test; b, *p* < 0.05 vs. control, Wilcoxon Paired test.

PDGF, FGF-1, and TGF-β are involved in the regulation of cell survival, growth, and apoptosis, and studies have shown their roles in ageing [25,26,27]. After six months of infection, patients with severe COVID-19 presented reduced PDGF (Figure 4B) and TGF-β (Figure 4J), as compared with controls, whereas the levels of FGF-1 were not different (Figure 4F). There were no differences in the blood levels of PDGF, FGF-1, and TGF-β when comparing the samples collected after six months of disease versus those collected late after recovery (Figure 4C,G,K). Nevertheless, when comparing samples from controls, after six months and late recovery, significant reductions in PDGF (Figure 4D) persisted, whereas no changes were detected in FGF-1 and TGF-β (Figure 4H,L).

## 4. Discussion

This study has shown that the blood levels of GH, FGF-1, BDNF, and PDGF are reduced after six months of COVID-19, and the reductions persist after fifteen months of recovery. All the hormones and growth factors evaluated in this study were selected because of prior reports of their roles in ageing, and because, to a certain degree, they are regarded as potential biomarkers of ageing [21,24,25,28]. The rationale of the study was that a considerable number of patients that develop severe COVID-19 may progress to show symptoms of late COVID-19, and this could predispose them to accelerated ageing.

The negative impact of infections on ageing has been shown in other contexts; however, in COVID-19, this association is still under investigation. Persistent infections can lead to chronic inflammation, which can, over time, damage tissues and accelerate the development of age-related conditions such as cardiovascular disease, arthritis, diabetes, and neurodegenerative disorders [29]. In addition, chronic infections can affect the immune system, impairing the proper functioning of both innate and adaptive arms of the immune response, and leading to an immunosenescence-like phenomenon [6].

In COVID-19, there is some evidence suggesting that the hyperinflammatory response that occurs during the acute phase of disease could trigger a pro-ageing programme that leads to cell damage, immune dysfunction, endothelial dysfunction, and telomere shortening, which in the long-run could contribute to an accelerated ageing process [30]. Moreover, it has been shown that COVID-19 can promote persistent damage to mitochondria, which is an important phenomenon taking place in ageing [31,32]. However, it was previously unknown if COVID-19 could promote persistent changes in the blood levels of hormones and growth factors involved in ageing.

The GH/IGF-1 system has been implicated in ageing in different ways. The first evidence for its pivotal role in this process came from an experimental work that promoted the knockout of orthologues of the system in the nematode *C. elegans* and resulted in increased longevity [33]. Further work showed that the effects of the genetic modulation of the GH/IGF-1 system could be, to a certain degree, reproduced by caloric restriction [34]. In this context, there was also a reduction in blood levels of insulin, which contributed to the development of the increased-longevity phenotype [29,30]. In humans, studies have shown that both GH and IGF-1 blood levels reduce with ageing [35]. This could seem controversial compared with experimental studies in which the genetic abrogation of components of the system promotes longevity; however, as in other hormonal systems, it is possible that optimal signals for controlling metabolism, health and, thus, longevity could work according to a hormetic pattern in which different levels of hormones could promote different outcomes [36]. Furthermore, research on untreated individuals with deletions in the GH gene, those experiencing multiple pituitary hormone deficiencies caused by PROP-1 gene mutations, and individuals with isolated IGF-I deficiency due to deletions or mutations in the GH receptor gene, despite displaying signs of premature ageing like skin wrinkles, obesity, insulin resistance, and osteopenia, indicates the emergence of a longer life expectancy, with some reaching ages between 80 and 90 years [37].

Moreover, the hypothalamic–pituitary–adrenal (HPA) axis has been identified as one of the most critical endocrine targets of severe COVID-19 and may significantly impact post-infection outcomes [38]. Several studies have reported hypothalamic and pituitary involvement potentially, associated with SARS-CoV-2 [38]. These studies mainly include cases of hypophysitis, hypopituitarism, pituitary apoplexy, inappropriate antidiuretic hormone secretion (SIADH), and diabetes insipidus [38,39,40,41]. The persistent condition observed after acute COVID-19 infection may be partly explained by deficiencies in the pituitary production of adrenocorticotropin (ACTH) and growth hormone (GH) [38]. Acute infection can lead to an intense inflammatory response, which can interfere with pituitary function, resulting in inadequate hormone secretion [38]. In our case, the reduction in, and persistence of decreased levels of, GH and IGF-1 could result in a phenotype similar to the one previously mentioned, suggesting that COVID-19 affects the GH/IGF-1 system in a manner akin to ageing, in addition to likely affecting the hypothalamic–pituitary–adrenal (HPA) axis.

BDNF and NGF are important growth factors involved in the development and preservation of the central and peripheral nervous system [36]. They act through two distinct receptors (TrkB and p75 for BDNF, and TrkA and p75 for NGF) that transduce signals that control both cell survival and apoptosis [23,42]. Perhaps because of the duality of functions exerted by BDNF and NGF, which depends on the locations and expression levels of their receptors, there are some controversies regarding the roles of these growth factors in ageing [28,43]. However, most studies suggest that reductions in BDNF and NGF occurring in the elderly may have a negative impact on cognition and promote other abnormalities of the nervous system that occur during ageing [28]. In our study, we showed that BDNF, but not NGF, undergoes a reduction after six months of the disease, and this reduction persists for at least fifteen months after recovery. Thus, BDNF may be involved in the regulation of ageing in COVID-19.

An interesting study demonstrated the association between serum BDNF levels and cognitive decline observed in a group of patients who had previously had mild COVID-19 [44]. The authors found a reduction in BDNF levels in patients who had persistent cognitive dysfunction compared to controls [44]. Another study observed decreased serum NGF levels in a cohort of children, but they showed negative results after COVID-19 infection. In this same study, BDNF levels only increased in symptomatic girls after COVID-19 infection. The presence of COVID-19-associated comorbidities at the time of sample collection is likely to influence circulating BDNF levels [45]. We believe that the interpretation of contrasting results (increased or decreased serum BDNF/NGF levels) is not clear and well defined.

It has been proposed that oxytocin is involved in ageing by at least two distinct mechanisms: the first is by protecting ageing people against the early loss of social instructiveness [46], and the second is by acting as a regenerative factor, predominantly in the muscle, and thus mitigating ageing-associated sarcopenia [47]. In this study, we could find no changes in the blood levels of oxytocin in patients with severe COVID-19.

Ghrelin is a hormone produced by the stomach during fasting [48]. Because caloric restriction is regarded as an important intervention leading to protection against ageing [34], ghrelin was proposed as a potential mediator of these effects. In this study, we could find no changes in the blood levels of ghrelin in patients with severe COVID-19.

PDGF is an important growth factor involved in tissue repair and regeneration [48]. Studies have shown that platelet-rich plasma has wound-healing properties and, in part because of that, PDGF was proposed as an anti-ageing factor [48]. These molecules are released by activated platelets and play a role in immune modulation and cell survival [49]. A study that blocked PDGF resulted in an increase in pro-inflammatory cytokines, while the administration of PDGF led to a decrease in these cytokines in patients with sepsis [50]. Another study found elevated levels of PDGF during COVID-19, indicating that this growth factor is involved in efforts to reduce the expression of pro-inflammatory cytokines [51]. Furthermore, PDGF signalling plays a crucial role in several age-related processes, including cellular senescence, immune function, and bone health [52,53]. In our study, we showed that PDGF undergoes a reduction after six months of the disease, and this reduction persists for at least fifteen months after recovery. This may be related to the exhaustion of this protein’s secretion by platelets, which diminishes this signalling pathway and could contribute to a premature ageing effect.

FGF-1 is involved in the regulation of several physiological processes, such as development, angiogenesis, wound-healing, adipogenesis, and neurogenesis [54]. The dysregulation of FGF-1 signalling is not only implicated in tumorigenesis but is also associated with tumour invasion and metastasis [54]. Experimental interventions that increasing FGF-1 expression can prevent high-fat-diet-induced obesity and insulin resistance and reduce fasting blood glucose and triglyceride levels by regulating lipolysis in adipose tissues and hepatic glucose production. Furthermore, increased FGF-1 expression can inhibit renal and cardiac fibrosis by regulating the expression of extracellular matrix components [54]. However, we did not identify changes in FGF-1 levels after six months of infection or after later convalescence when compared to the control. There are few studies in the literature reporting FGF-1 modulation during COVID-19 and long-term COVID-19. Nonetheless, some previous studies corroborate our data. One study, involving 210 patients diagnosed with COVID-19 and 80 healthy individuals, evaluated the plasma concentrations of several vascular factors, including FGF-1. The researchers found no statistically significant differences in FGF-1 concentrations between the groups [55]. Another study with COVID-19 patients found no significant correlation between the levels of FGF23 [56] (which belongs to the FGF family) and several classical inflammatory markers [56]. The lack of modulation of this gene may represent a favourable aspect concerning long-term COVID-19, as the increased expression of FGF-1 is associated with various metabolic defects and tumorigenesis.

TGF-β is a crucial cytokine in the regulation of essential biological functions and it is implicated in the onset and progression of multiple diseases, such as several types of cancer, inflammatory and immunological diseases, heart disease, pulmonary and renal fibrosis, and neurodegenerative diseases [43,48,57,58]. Studies show that in post-COVID-19 survivors, TGF-β1 is associated with a high incidence of fibrosis in individuals who survived severe acute respiratory syndrome caused by the disease. This association is influenced by factors such as lung injury and an exaggerated response to tissue damage [59,60,61]. Alfaro et al. indicated that in fatal cases of COVID-19, TGF-β1 may have both anti-inflammatory and pro-inflammatory effects, contributing to endothelial damage [62]. Frischbutter et al. demonstrated significantly reduced TGF-β1 levels in critically ill patients treated with dexamethasone compared to untreated patients [63]. Zivancevic-Simonovic et al., in a study involving 53 patients with severe COVID-19 and 15 control subjects, measured serum TGF-β1 concentrations, and lower serum values were associated with an unfavourable disease outcome [64]. However, TGF-β1 production needs to be activated by proteases to promote profibrotic signals, increasing the accumulation of proteins and other components of the extracellular matrix [65]. In our study, we evaluated the active and inactive forms of TGF-β1 and observed that TGF-β1 levels were decreased after 6 months of COVID-19 compared to the control group, and these levels remained decreased after 15 months of the disease. The lower levels of TGF-β1 found in our study may have been due to the treatment with dexamethasone administered to these subjects during hospitalization, as noted in the study by Frischbutter et al. [63].

## 5. Conclusions

This is the first study showing that the blood levels of GH, IGF-1, BDNF, and PDGF are reduced during acute severe COVID-19 and that the reduced levels persist for at least six months after recovery. These hormones and growth factors play significant roles in the ageing process. The GH/IGF-1 system is involved in promoting longevity and regulating metabolism, while BDNF is associated with cognitive decline in patients who have had COVID-19. Additionally, PDGF is a factor linked to anti-ageing, including cellular senescence, immune function, and bone health. The data found in this study advances the field by identifying hormones and growth factors that can easily be determined in blood samples; these might act of biomarkers of the accelerated process of ageing in COVID-19, as well as potential therapeutic targets for approaches aimed at mitigating the long-term damage promoted by the infection.

## Figures and Tables

**Table 1:** Comorbidities in patients and control subjects.

	*Group*				*p-Value*
	Control		COVID-19/1º		
	n	%	n	%	
Sex					0.5448 *
*Male*	14	50	28	57.14	
*Female*	14	50	21	42.86	
*Comorbidities*					0.2027 *
*Yes*	8	28.57	8	16.33	
*No*	20	71.43	41	83.67	
*Obesity*					0.9656 *
*No*	13	46.43	23	46.94	
*Yes*	15	53.57	26	53.06	
*Hypertension*					0.0634 *
*No*	11	39.29	30	61.22	
*Yes*	17	60.71	19	38.78	
*Dyslipidemia*					0.0003 *
*No*	16	57.14	45	91.84	
*Yes*	12	42.86	4	8.16	
*Diabetes*					1.0000 *
*No*	20	71.43	35	71.43	
*Yes*	8	28.57	14	28.57	
*Asthma*					1.0000 **
*No*	26	92.86	45	91.84	
*Yes*	2	7.14	4	8.16	
*Respiratory disease*					0.5311 **
*No*	28	100	47	95.92	
*Yes*	0	0	2	4.08	
*Hypothiroidism*					0.1937 **
*No*	22	78.57	44	89.8	
*Yes*	6	21.43	5	10.2	

COVID-19/1º, coronavirus disease-19. * chi-square; ** Fisher’s exact test.

## Data Availability

Data will be available upon request directed to the corresponding author, E.P.A. (earaujo@unicamp.br) and available at the Data Repository of the State University of Campinas-REDU.

## Supplementary Materials

The following supporting information can be downloaded at: https://www.mdpi.com/article/10.3390/diseases13070209/s1, Table S1: Admission levels for serum interleukin-6 and interleukin-1 beta. The values are presented as mean ± standard deviation, along with the normality values for each parameter. Table S2. General characteristics of patients and control subjects. BMI, body mass index; COVID-19/1º, coronavirus disease-19; SD, standard deviation. * Unpaired Student’s *t* test; ** Mann-Whitney.

## Abbreviations

The following abbreviations are used in this manuscript:

AG	Acylated Ghrelin
BDNF	Brain-Derived Neurotrophic Factor
CNPq	National Council for Scientific and Technological Research and Development
COVID	Coronavirus Disease
FGF-1	Fibroblast Growth Factor-1
GH	Growth Hormone
IGF-1	Insulin-Like Growth Factor-1
NGF	Nerve Growth Factor
OCRC	Obesity and Comorbidities Research Center
OT	Oxytocin
PCR	Polymerase Chain Reaction
PDGF	Platelet-Derived Growth Factor
SARS-CoV-2	Severe Acute Respiratory Syndrome Coronavirus 2
TGF-β	Transforming Growth Factor-Beta
Trka	Tropomyosin Receptor Kinase A
Trkb	Tropomyosin Receptor Kinase B
UNT	Universal Trial Number
