# Supplementary material for: Persistent Changes in Hormones and Growth Factors Involved in Ageing in Patients That Recovered from Severe COVID-19
Source: Diseases. 2025 Jul 3;13(7):209. doi: 10.3390/diseases13070209 (PMC12294000; doi:10.3390/diseases13070209)
Supplement: Supplementary file 1 [file diseases-13-00209-s001.zip › diseases-3696591-supplementary-done.pdf]

diseases

MDPI

Supplementary data

Table 1. Admission levels for serum interleukin-6 and interleukin-1beta.

Parameter	Value	Normality value
Serum Interleukin-6	26.3±25.1 pg/mL	7-15 pg/mL
Serum Interleukin-1 beta	87.2±97.4 pg/mL	0.5-12 pg/mL

Table 2. General characteristics of patients and control subjects.

	Group	n	Mean	SD	p-value
Age (y)	Control	28	52.96	12.03	0.8640*
	COVID-19	49	53.47	12.61	
Body mass (kg)	Control	28	86.86	20.14	0.5080**
	COVID-19	49	91.07	22.84	
BMI	Control	28	30.81	6.11	0.6187**
	COVID-19	49	31.86	6.94	

BMI, body mass index; COVID-19/1º, coronavirus disease-19; SD, standard deviation. * Unpaired Student´s t test; **Mann-Whitney.
